# Comment on ‘polygamy, sexual behavior in a population under risk for prostate cancer diagnostic: an observational study from the black sea region in Turkey’

**DOI:** 10.1590/S1677-5538.IBJU.2018.0459

**Published:** 2018

**Authors:** Mete Özkidik, Anar İbrahimov

**Affiliations:** 1 Department of Urology, Sanliurfa Egitim ve Arastirma Hastanesi, Karşiyaka, Şanliurfa, Turkey; 2 Ankara university, Ankara, Turkey

*To the editor,*

I read the article by abdullah cirakoglu et al. (1) with great interest but i have some points of concern with this article.

Firstly, it is stated that all patients participated in the study have similar lifestyle and nutritional habits. However, the details of lifestyle and nutritional habits such as having office or night - shift work, consumption of processed meat or alcohol and smoking habits were not mentioned in the article. Also using hormonally active medications would be a predisposing factor for prostate cancer. Testosterone supplements (2) or 5 - alpha - reductase inhibitors (5 - ARIs) (3) are common examples currently under research whether leading to prostate cancer. The article does not reveal any details about the routine medications of the participants. On the other hand, I think, it would be rarely possible to say that all of 317 patients who were assessed in the study have similar lifestyles and nutritional habits even they used to live in the same geographical region.

Secondly, the increased number of sexual partners is under consideration in the article that it might be liable for having prostate cancer. I think it would be an important predisposing factor for prostate cancer due to causing chronic prostatitis which leads to prostatic intraepithelial atrophy (PIA) and than cancer in the long - term by recurrent sexually transmitted infections (STIs) (4). But the history of urethritis or prostatitis of the participants were not mentioned in the article. Although serologic testing could not be performed, questioning the history of urethritis would be beneficial. So, I think that considering only the number of sexual partners of the participants through their whole life would not be appropriate to determine the risk of prostate cancer unless the number or severity of attacks of prostatitis is considered.

Finally, sexual intercourse frequency is assessed in the study population as a risk factor for prostate cancer. Sexual intercourse frequency per month is compared between the two groups only for the youth and current period. But the limits of age for these periods are not determined in the article. In other words, it is not clear which decade is referred as considering “youth” or “current”. In addition, there is conflicting data about the association of ejaculation frequency (5) and prostate cancer in the current urologic literature. For some authors, the age of 30 is a critical point that ejaculation frequency in younger ages and older ages have different impacts on development of prostate cancer. So I believe that the period of age should also be mentioned in the article as well as the frequency of sexual intercourse as considering the risk of prostate cancer.
